# Dr. Charles H. North

**Published:** 1918-02

**Authors:** 


**Dr. Charles H. North, Buffalo** 1898, died in the discharge of duty at the Dannemora State Hospital for Insane Felons, Dec. 12, aged 49, being stabbed by one of the inmates.

